# Naringin: Nanotechnological Strategies for Potential Pharmaceutical Applications

**DOI:** 10.3390/pharmaceutics15030863

**Published:** 2023-03-07

**Authors:** Soledad Ravetti, Ariel G. Garro, Agustina Gaitán, Mariano Murature, Mariela Galiano, Sofía G. Brignone, Santiago D. Palma

**Affiliations:** 1 Instituto Académico Pedagógico de Ciencias Humanas, Universidad Nacional de Villa María, Villa María 5900, Argentina; 2 Centro de Investigaciones y Transferencia de Villa María (CIT VM), Villa María 5900, Argentina; 3 Ministerio de Ciencia y Tecnología, Gobierno de Córdoba, Córdoba 5004, Argentina; 4 Inbiomed-Ingeniería Biomédica, Córdoba 5010, Argentina; 5 Unidad de Investigación y Desarrollo en Tecnología Farmacéutica (UNITEFA), CONICET, Departamento de Ciencias Farmacéuticas, Facultad de Ciencias Químicas, Universidad Nacional de Córdoba, Córdoba 5000, Argentina

**Keywords:** natural products, naringin, nanoformulation, bioavailability, drug delivery

## Abstract

Polyphenols comprise a number of natural substances, such as flavonoids, that show interesting biological effects. Among these substances is naringin, a naturally occurring flavanone glycoside found in citrus fruits and Chinese medicinal herbs. Several studies have shown that naringin has numerous biological properties, including cardioprotective, cholesterol-lowering, anti-Alzheimer’s, nephroprotective, antiageing, antihyperglycemic, antiosteoporotic and gastroprotective, anti-inflammatory, antioxidant, antiapoptotic, anticancer and antiulcer effects. Despite its multiple benefits, the clinical application of naringin is severely restricted due to its susceptibility to oxidation, poor water solubility, and dissolution rate. In addition, naringin shows instability at acidic pH, is enzymatically metabolized by β-glycosidase in the stomach and is degraded in the bloodstream when administered intravenously. These limitations, however, have been overcome thanks to the development of naringin nanoformulations. This review summarizes recent research carried out on strategies designed to improve naringin’s bioactivity for potential therapeutic applications.

## 1. Introduction

Polyphenols comprise a number of naturally occurring and biologically-active substances, including flavonoids. Flavonoids are an important group of secondary metabolites and a permanent source of compounds with a wide spectrum of biological actions that may be able to stimulate steps altered in different diseases. Citrus flavonoids establish an important stream of flavonoids, with naringin (NRG) standing out in this classification [1].

NRG was first discovered in grapefruit flowers by De Vry in 1857, however, the results of his research were not published at that time [2]. NRG (4′,5,7-trihydroxy flavanone 7-rhamnoglucoside) is a naturally occurring flavanone glycoside present in several plant species, and it is one of the most widely used active compounds in Chinese herbal medicine. Subsequent research reported that NRG accumulates at a high level in various citrus fruits, such as grapefruit and sour orange. The characteristic bitter taste of citrus juices is produced due to the accumulation of NRG.

**Figure 1:**
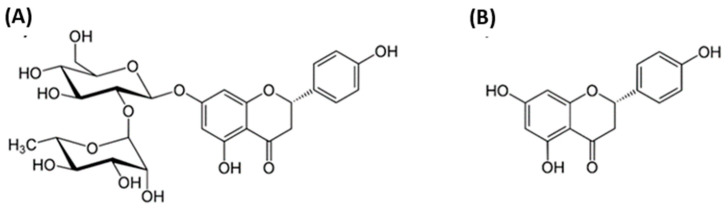
Chemical structures of naringin (NRG) (**A**) and naringenin (NRGN) (**B**).

Figure 1A shows the structure of NRG, which consists of flavanone naringenin (NRGN), Figure 1B) and disaccharide neohesperidose linked through a glycosidic bond. The molecular formula of NRG is C_27_H_32_O_14_ and its molecular weight is 580.53.

**Figure 2:**
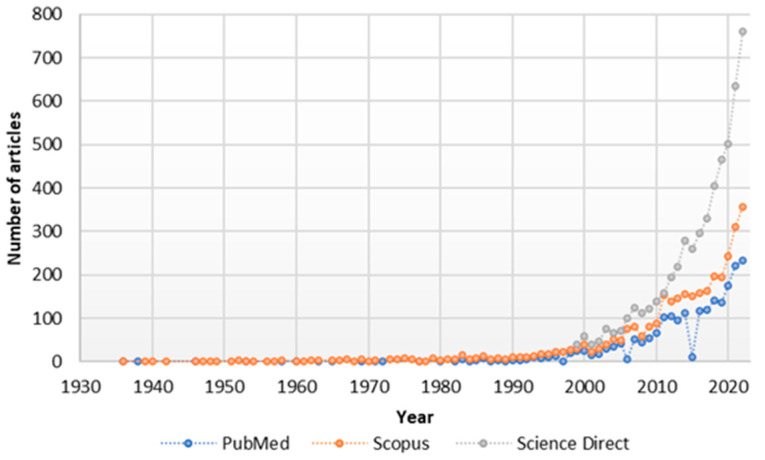
Number of Publications by Year/Database.

Since 2010, there has been a marked scientific interest in NRG, as can be clearly seen in Figure 2, which shows an increasing number of articles published with the keyword naringin in different databases (Science Direct, Pubmed, and Scopus).

Based on this research, a large number of scientific articles have been conducted on NRG describing its structure, physicochemical properties, and its therapeutic uses in different diseases.

The concentration of NRG in fruit is dependent on numerous factors, such as the time of fruit collection, the part of the fruit analyzed, whether the peel is the reservoir of NRG, and the fruit’s drying time [3].

The process of obtaining NRG from fruits is similar to that of other flavonoids such as hesperidin, and it basically consists of three steps: extraction, separation, and purification.

Maceration, percolation, thermal reflux, Soxhlet, supercritical, microwave assisted, and ultrasound assisted are some of the often-employed methods of NRG extraction [4].

**Figure 3:**
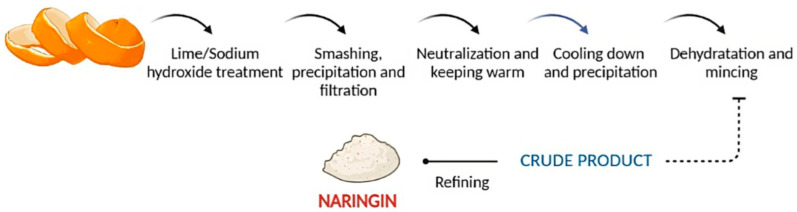
Steps in the naringin alkaline extraction process.

One of the most conventional methods applied, used for its simplicity, is the extraction of NRG with an alkaline solution of pH 9–10, such as lime or sodium hydroxide, and its posterior precipitation in cold acidic water. Figure 3 shows, representatively, the steps necessary to isolate NRG.

Kanokorn et al. have developed an efficient and simple method to isolate high-purity NRG from grapefruit of Citrus grandis Osbeck peel in high yield. The described process, consisting of a simple extraction with methanol followed by crystallization in water with the addition of 14–15% (*v*/*v*) dichloromethane, gave a yield four times higher compared to the same method without the addition of dichloromethane and five times higher than compared to conventional direct extraction with hot water. The yield obtained from different cultivars was 16–24 mg per gram of dry weight of peel [5].

Within the group of techniques for the identification and purity control of NRG, there can be mentioned ultraviolet-visible spectroscopy (UV-VIS), Fourier transform infrared spectroscopy (FTIR), nuclear magnetic resonance spectroscopy (NMR), mass spectrometry (MS), and elemental analysis (EA) [6].

## 2. Bioavailability and Pharmacokinetic Properties of Naringin

NRG is slightly soluble in water (1 mg/mL at 40 °C) [7]. In organic solvents, NRG shows a higher solubility in polar solvents (methanol > ethanol > ethyl acetate) compared to nonpolar solvents (petroleum ether > hexane), probably due to the hydrophilic sugar residues in its structure.

The solubility of NRG in different solvents also becomes more plausible with increasing temperatures in certain ranges [8].

However, according to the Biopharmaceutical Classification of Drugs (BCS), NRG is a class IV drug, which indicates that it is a compound with significant limitations for oral administration. Lack of solubility and low permeability result in a limited bioavailability of NRG due to its large hydrophobic ring structure [9].

The degree of conjugation with sugar moieties [10,11,12] and its corresponding elimination by intestinal bacterial species are directly related to NRG metabolism. In addition, NRG is unstable at acidic pH and enzymatically cleaved by β-glycosidase in the stomach [13,14].

NRG is also resistant to enzymatic attacks in the stomach and small intestine, so it will eventually reach the colon.

Oral absorption of NRG from citrus products suggested that NRG is poorly absorbed from the gastrointestinal tract in its original form [15] and it is often exposed to enzymes secreted by the microbiome of the gut region. The process begins with the elimination, first of rhamnose, and then of glucose via enzymes β-glucosidases.

**Figure 4:**
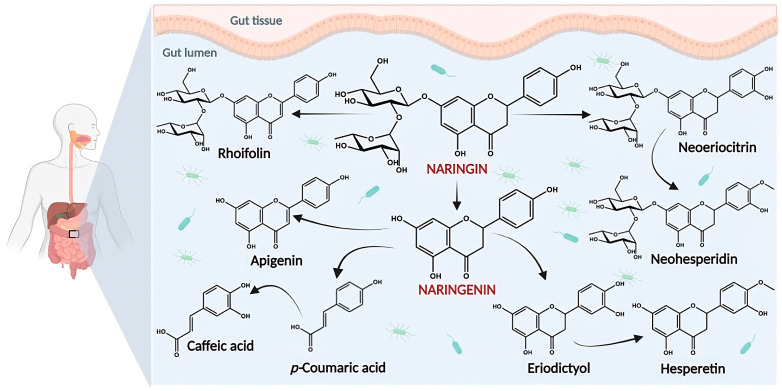
Some potential pathways for the metabolism of NRG by the gut microbiota.

As shown in Figure 4, NRG is broken down into its aglycone NRGN in the gut by intestinal microflora, a process which further affects its bioavailability [16], metabolizes it into other flavonoid metabolites, and degrades it into a variety of ring fission products [16,17,18].

Recent studies have demonstrated the important role of these microbial metabolites in the overall bioactivity of NRG [19,20]. In fact, the physiological effects of NRG cannot be fully achieved in the absence of microbial metabolites due to the extensive biotransformation of the parent compound [21]. Although significant conversion takes place in the colon, some breakdown also occurs in the distal portion of the small intestine [22].

During its release, NRG is absorbed through the intestinal epithelium by passive diffusion and proton-coupled active transport. It is further metabolized into phenolic acids by the cleavage of the pyran ring, demethylation, and dehydroxylation with the help of bacterial enzymes.

It is therefore important to outline the intestinal microbial metabolism of NRG.

The free form of NRG can be found transiently in the plasma of rats and humans, and NRGN glucuronide appears to be the predominant metabolite [23,24]. It has been described that NRG is rapidly absorbed in a first concentration peak at 15 min and in a consequent one at 3 h after oral administration of monomeric NRG. Following 8 h of dosing, the authors found no evidence of rapid metabolism (5.075 mg/kg). The area under the curve (AUC) and the mean residence time (MRT) resulted in 274.8 mg/L and 114.0 min, respectively [25].

## 3. Biological Activities of Naringin

NRG has been explored broadly for its biological and pharmacological effects. Most of the health benefits of NRG are related to the common chemical structure of natural flavonoids and to the presence of the sugar moieties and hydroxyl groups attached to both aromatic rings, structures that provide particular physicochemical and physiological characteristics able to perform numerous functions and distinguish themselves from others. It has been recently demonstrated that NRG exerts potential therapeutic actions by modulating various protein and enzyme expressions [26].

**Figure 5:**
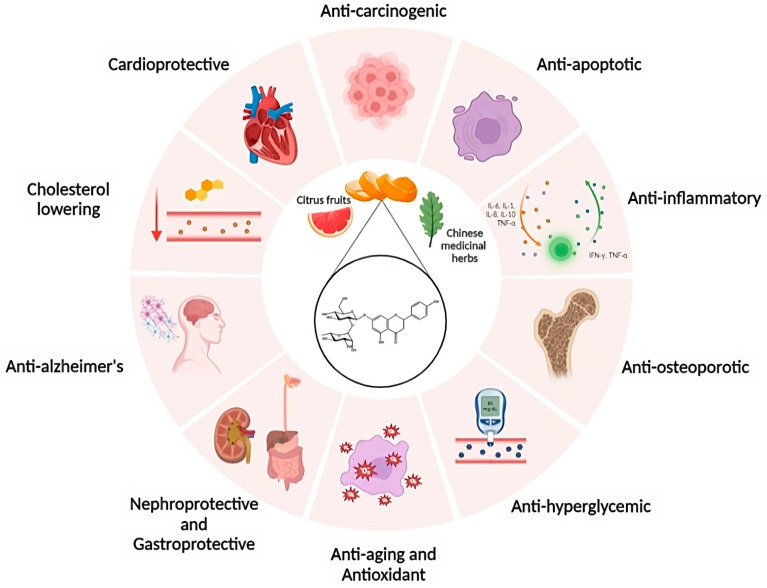
Health effects of naringin on different biological systems.

As shown in Figure 5, NRG has been documented to possess antioxidant, neuroprotective, anti-inflammatory, antiapoptotic, antiulcer, antiosteoporotic, and anticancer properties [27,28,29,30,31,32].

In recent years, NRG has also been increasingly used as a phytopharmaceutical in dietary supplement formulations [33].

## 4. Clinical Translation and Challenges for Its Therapeutic Application

Despite its benefits in the prevention and treatment of various diseases [34,35], NRG has not yet been approved for clinical administration either as a single therapy or in combination with other bioactive compounds, mainly due to the intense in vivo metabolism of flavonoids that restricts its therapeutic efficacy [34,35].

In addition, the low solubility and dissolution rate of NRG has resulted in low bioavailability (approximately 8.8%) when administered orally. The limiting step in its absorption in the organism is caused due to the poor water solubility of NRG, which results in lower therapeutic efficacy [15,36].

Furthermore, this specific drug degrades in an acidic pH environment and is easily metabolized in the intestinal region by the enzyme β-glucosidase, an inherent property of the intestinal microflora [37].

In most cases, significant exploration of NRG occurs mainly through the oral route, however, its absorption in the intestinal tract does not take place in the same manner. In comparison, NRG’s absorption in the GIT tends to be rather slow and erratic [13].

In addition, the microbes present in the gut play an important role in the bioavailability and clinical efficacy of flavonoids such as NRG [38].

Currently, several efforts have been concentrated on overcoming certain disadvantages of NRGs for use in clinical applications. In vitro attempts have been made to improve the bioavailability and absorption of flavonoids by modifying their solubility and dissolution rates along with the prevention of their degradation due to gut microbes, remarkably through the encapsulation of either nanoparticles or microparticles [13,38,39].

When administered intravenously (IV), NRG can also degrade during blood circulation. This structure is generally not stable when present in the bloodstream and readily undergoes oxidation in the serum and liver, where it is degraded by β-glucosidases [37].

According to the above report, NRG interacts with bovine serum albumin immediately under physiological conditions defining its pharmacokinetic profile, which promotes excretion and thus influences the bioavailability of NRG [40].

Various strategies are often proposed to improve the bioavailability and bioactivity of biologically active compounds for medicinal purposes. These strategies include advances in drug delivery systems from nanotechnology, pharmaceutical technology, and colloidal systems, as well as chemical structural modifications of a drug candidate, use of appropriate bioenhancers, and inhibition of intestinal cell transporters, among others.

The development of micro- and nanoformulations is recognized as one of the most potent lines of research to overcome the physicochemical and biopharmaceutical limitations of drugs in the treatment of pathologies and diseases. Numerous micro- and nanotechnology-based drug delivery systems have improved the bioavailability and pharmacokinetics of NRG, which ultimately protects it from degradation and random interactions when it has prolonged circulation times [41,42].

These systems have the potential to exhibit a sustained release profile after appropriate modification with precise targeting of elements to enhance accumulation at specific sites [43]. Thus, it is possible to improve the bioavailability of NRG by conjugating it to a suitable micro- or nanotransporter [44].

## 5. Naringin Nanoformulations

**Figure 6:**
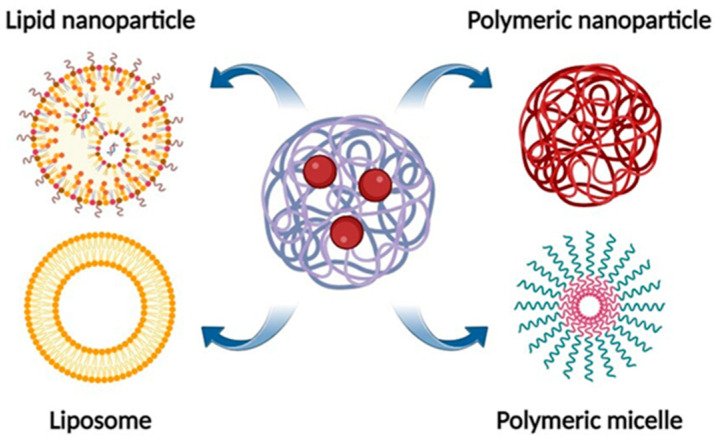
Naringin-loaded nanoformulations for drug delivery systems.

As described in Figure 6, in recent years, the amount of research focused on incorporating NRG into various nanocarriers to enhance its bioactivity in different biological systems has increased considerably.

These structures include liposomes, micelles, nanocrystals, solid lipid nanoparticles (SLNs), scaffolds, and nanostructured lipid carriers.

From the literature reviewed, numerous systems have demonstrated significant advantages over pure NRG in different therapeutic applications, although information on the in vivo performance of these NRG-loaded nanoparticles is still lacking.

The most recent nanoformulations (liposomes, polymeric micelles, polymer-based nanoparticles, and lipid nanoparticles) designed for the delivery of NRG by different routes of administration are described below.

### 5.1. Liposomes

Liposomes are one of the most evaluated drug delivery systems for therapeutic purposes due to their versatility to incorporate drugs or other substances with hydrophobic, hydrophilic, and even amphiphilic characteristics. Depending on the method of preparation, these colloidal vesicles range in size from 20 nm to several microns. As drug delivery systems, they are used in all types of liquid, solid, and semisolid formulations, and for different routes of administration, including oral, intramuscular, subcutaneous, ocular, and intravenous.

However, very few liposomal systems have shown promising results for the transport of NRG for therapeutic purposes. Among them, formulated by Pleguezuelos-Villa et al., are ultradeformable liposomes loaded with NRG for skin inflammatory therapy [45]. These liposomes have already shown to be promising nanosystems for the improvement in the cutaneous delivery of herbal extracts whose deformability and elasticity are attributed to the combination of phospholipids and surfactant, where the surfactant acts as an edge activator that modifies the organization of lipid bilayers and increases their deformability. The authors obtained monodisperse nanoliposomes of small size (∼100 nm) with a high entrapment efficiency (EE) of NRG (∼88%) and a negative zeta potential. In vitro studies with 3T3 mouse dermal fibroblast cells showed them to be biocompatible, although the permeability tests (24 h) performed indicated that the percentage of NRG accumulated in the epidermis is low (∼10%), suggesting low permeability. The authors attribute that this amount of accumulated NRG would be responsible for the inflammatory properties detected in the in vivo model (TPA test), being more effective than the results obtained after the application of betamethasone cream. They concluded that NRG loaded with ultradeformable liposomes can be considered a promising formulation for the treatment of skin inflammatory diseases.

In another recent publication, Mohanty et al. (2020) describe the therapeutic potential of novel liposomal formulations loaded with NRG and other nutraceuticals (isothiocyanates) with promising antiinflammatory properties for the treatment of rheumatoid arthritis (RA) [46]. These authors prepared two combinations of liposomes (NRG + sulforaphane (SFN) and NRG + phenethyl isothiocyanate (PEITC)) and studied their behaviors in different in vivo models of acute and chronic inflammation. They used DSPE-020CN (N-(carbonyl-methoxy polyethylene glycol 2000) DSPE, sodium salt), which only differs from DSPE-PEG2000 by a methoxy (−OCH_3_) group versus an amine (−NH_2_) group attached to the polyethylene glycol (PEG) chain. Their purpose was to modify the liposomal surface and to achieve a longer circulation time of the formulation, which would probably allow a greater accumulation in the inflamed area due to the ELVIS effect (extra due to vascular leakage and subsequent sequestration mediated by inflammatory cells).

The different formulations obtained present a low mean particle size range (140.5 to 165.6 nm), and monodisperse (PDI 0.062 − 0.248) with a highly negative surface charge distribution (−47.3 to −53.3 mV). The authors point out that liposomes prepared from the 15:4:1 M ratio of DPPC/Chol/DSPE-020CN are the most efficient for the encapsulation of the different compounds, which is why these systems, charged with different combinations of liposomal formulations (15:4:1 NRG + SFN and 15:4:1 NRG + PEITC) were used for in vivo studies. In both models of inflammation (acute and chronic) induced in rats, these liposomal formulations showed promising results: Both NRG + SFN and NRG + PEITC formulations were able to significantly decrease proinflammatory cytokine levels, and increase anti-inflammatory cytokines, reduce the edema produced by the subcutaneous injection of egg albumin as well as the edema induced by carrageenan, elevate the C-reactive protein (CRP), reduce serum rheumatoid factor (RF) levels to a normal range, aid in the diminishment of the levels of oxidative stress biomarkers such as glutathione (GSH), superoxide dismutase (SOD), and serum catalase, and significantly inhibit acute joint destruction with reduced granulocyte infiltration. It is due to the slower release profile observed in in vitro studies that the authors indicate that NRG + PEITC liposomes are more effective than NRG + SFN liposomes. Both PEITC and NRG are released for more hours (6 h), implying that they would remain longer in the body compared to SFN, which is released for 3 h.

NRG-loading liposomes have also been designed as a potential platform for the treatment of pulmonary fibrosis through inhalation delivery. In this case, Kotta et al. (2021) used phosphatidylcholine as endogenous pulmonary surfactant-mimetic lipids to prepare the liposomes and to be administered as an aerosol [47]. The use of pulmonary surfactant-mimetic lipids would help to reduce the surface tension associated with mechanical stress in the alveolar interface and to prevent alveolar collapse, also favoring deep lung delivery.

The phosphatidylcholine-based liposomes obtained by this group showed good EE of NRG, a spherical unilamellar structure in the nanometer range, and negative zeta potential. These liposomes exhibited efficient surfactant function in addition to acting as a pulmonary drug delivery system. Airway patency studies in vitro indicated that the formulation maintained airway patency of 97 ± 2.5% for 120 s, ensuring deep lung delivery. The most promising results were those obtained in an in vivo model of bleomycin-induced pulmonary fibrosis. Treatment with liposomal NRG reduced inflammatory cell infiltration in the bronchoalveolar lavage fluid, as well as lactate dehydrogenase enzyme activity, total protein content, and oxidative stress. In addition, staining of the lung tissue revealed a significant improvement in histological changes and less collagen deposition. Based on these results, the authors postulate these NRG-loaded phosphatidylcholine-based liposomes as a viable approach for the treatment of pulmonary fibrosis, since they offer the beneficial effects of NRG on pulmonary fibrosis [48] added to the contribution of the liposomal system that allows poor access to deep alveoli for a better therapeutic effect. Taking this information into account, the application of these structures could even be extended to other lung diseases.

Zheng et al. (2022) designed the transactivator of transcription (TAT) peptide and arginine-glycine-aspartate (RGD) tripeptides-modified NRG-loaded liposomes to promote the osteogenic properties of NRG [49]. The TAT peptide, derived from the human immunodeficiency virus (HIV) transcription protein, is part of a group of short peptides classified as cell-penetrating peptides (CPPs), widely used as ligands to facilitate intracellular uptake [50]. The RGD peptides are recognized as specific ligands for integrin transmembrane receptors, which are key regulators of cell–cell communication and the cell–extracellular microenvironment [51]. NRG loaded-liposomes were obtained using a film hydration method and extrusion filtration with 400 nm, 200 nm, 100 nm, and 80 nm filter membranes, and subsequently stored at 4 °C. Afterwards, the TAT and RGD peptides were conjugated by previously activating the carboxyl group of the NGR-liposomes using 1-ethyl-3-[3-dimethylaminopropyl] carbodiimide hydrochloride (EDC). The reported results show an average particle size of 160.70 nm, a negative zeta potential (−20.77 mV), and a low EE (6.82%). The NRG release from TAT-RGD-NRG-liposomes by the dialysis method was 38.1 ± 1.8% at 12 h and 63.9 ± 2.2% at 36 h. The reported in vitro assays revealed that the TAT-RGD-NRG-liposomes turn out to be more effective for the NRG administration on human dental pulp stem cells (hDPSCs) without causing cytotoxicity. In addition, the developed formulation produces a more significant effect in promoting cell proliferation and osteogenic differentiation, which is evidenced by the higher level of alkaline phosphatase (ALP) expression and the increase in the expression of genes related to osteogenesis as well as a higher degree of mineralization in hDPSCs.

Some reports also propose the use of new generations of liposomes for the topical and transdermal administration of NRG, such as ethosomes, proposomes, and phytosomes. These new liposomal generations are considered to be highly successful delivery systems for topical and transdermal routes of administration, mainly due to their elastic and deformable structures that improve drug penetration through the skin.

Gollavilli et al. (2020) designed NRG-loaded ethosomes to enhance the penetration and retention of this natural bioactive in the skin. In this way, NRG could be used by virtue of its photoprotective and antioxidant properties [52]. Ethosomes are liposomes that contain ethanol molecules as cosurfactant, which are able to be inserted into the bilayers in the region close to the polar heads of the phospholipids, making the vesicles less rigid [53]. In addition to making them more elastic and malleable, the presence of ethanol in their structure acts by modifying the conformation of the stratum corneum (SC) by altering its lipid organization, and, as a result, decreasing the fluidization of the SC and allowing greater penetration of the encapsulated drug until the deeper layers of the skin. NRG-loaded ethosomes were prepared, optimized, and then incorporated into sunscreen creams with nano-ZnO and -TiO_2_ as sunscreen agents. Ethosomes with better particle size and EE were obtained by the mechanical dispersion method. Skin permeation profiles revealed a higher permeation of NRG when formulated in the ethosomes compared to NRG suspension and creams. In addition, the NRG ethosomes showed higher retention in the skin (403.44 ± 15.33 μg/cm^2^) compared to the NRG suspension (202.81 ± 9.45 μg/cm^2^). This formulation also demonstrated the antioxidant activity of encapsulated NRG ((2,2′-azino-bis(3-ethylbenzothiazoline-6-sulfonic acid)) ABTS+ activity), although this was lower compared to standard curcumin and NRG suspension, probably due to slow NRG release from the ethosomes. Moreover, the optimized sun protection cream, particularly SC4, showed enhanced NRG retention within the skin with minimal permeation, as well as excellent sun protection factor (SPF) value. The authors also suggest that the ethosomes incorporated into sunscreen creams may be responsible for the stability of the prepared creams. This is the first study that shows the potential of using NRG-loaded ethosomes for skin applications.

Other authors designed NRG-loaded proposomes (propylene glycol (PG)-based liposomes) to improve wound healing and combat the effect of free radicals on the tissue healing process [54]. Free radicals (ROS) are critical factors that have a deep influence on the healing process, and NRG has shown properties able to inhibit the cell damage these induce.

In general, proposomes are used for the epidermal, dermal, and transdermal delivery of skin drugs and other therapeutic applications by varying their lipid and PG composition [55,56]. Like ethanol in ethosomes, PG is added to improve drug and formulation stability, which has as a result enhanced penetration and drug deposition on the skin. In this case, the authors loaded the NRG proposomes into a topical gel based on carbopol 974 polymer to achieve the best tissue regeneration. NRG-loaded proposomal gels were prepared from the hot microemulsion technique, characterized, and evaluated both in vitro and in vivo for different wound healing properties. The validation results of the optimized NRG proposomes show very good EE with a net negative surface charge (from −79.46 to −97.30 mV), which favors the stability of the formulation. The size remains in the nanometric range, though with a very wide polydispersity index (PI) (0.362 to 0.896). The NRG release profile from proposomes shows sustained release over 72 h. Regarding the formulated proposomal gel, it shows good properties such as an adequate pH for application to the skin, good spreadability of the gel, acceptable limits of NRG content, and a better release profile of NRG compared to topical gel during 96 h. The authors suggest that the gel controls NRG release and holds the compound at the site, making NRG available at the application site or on the surface of deep wounds. NRG loaded in the proposomal gel demonstrates adequate antioxidant activity in vitro, and most notably, it also demonstrates better wound closure on day 15 (3.3%) compared to the proposomal solution (4.8%) or topical NRG gel (4.2%). These results allow the postulation of this formulation as a potential strategy for rapid wound healing.

### 5.2. Polymeric Micelles

Micelles are generally spherical structures of nanometer size that are formed by the self assembly of different amphiphilic molecules. Micelles can improve the pharmacokinetics and tissue distribution of drugs, improve their bioavailability, provide sustained and controlled release of compounds, provide physical and chemical stability of encapsulated molecules, and allow intravenous administration [57].

Mohamed et al. (2018) designed NRG-loaded polymeric micelles based on pluronic F68 (PF68) as a strategy to enhance the antiulcer and anticancer activity of NRG [58].

Pluronics polymers not only have the ability to form micelles with a hydrophobic polypropylene oxide (PPO) core within a hydrophilic polyethylene oxide (PEO) shell in aqueous solutions but have also been used potentially to counteract tumor multidrug resistance (MDR) to numerous anticancer agents. Micelles prepared with the highest polymer content (NRG-PF68 1:50) showed the highest EE, a mean diameter smaller than 100 nm, a narrow size distribution, and good stability, which are the optimal conditions for a drug release system. Regarding the anticancer activity of the formulation, in vitro cytotoxicity studies showed that the NRG encapsulation in PF68 pluronic micelles significantly enhances its cytotoxicity against the different cell lines evaluated, with Caco-2 cells being the most sensitive. It even demonstrated better activity than the reference anticancer cisplatin. In vivo antitumor activity tested in Ehrlich ascites carcinoma (EAC)-bearing mice also showed greater inhibition of tumor growth in the group treated with the micellar NRG formulation compared to free NRG. The authors suggest that the enhancement of NRG cytotoxicity in vitro is due to micellization of NRG with pluronic polymers, responsible for increased penetration and retention in cancer cells as well as the potential inhibition of drug efflux pump or P-glycoproteins by pluronics. The authors also describe the comparative advantages of micellar NRG over free NRG ethanol-induced ulceration in a rat model. The micellar formulation produces a significant reduction in mucosal damage, decreases the gastric level of malondialdehyde and the gastric expression of alpha tumor necrosis factor, caspase-3, and favors the increase of gastric reduced glutathione and superoxide dismutase compared to the positive control group.

Jabri et al. (2018) also developed polymeric micelles as an effective delivery system for the codelivery of NRG and paclitaxel (PTX) in order to improve the anticancer efficacy of PTX in breast cancer [59]. This coadministration strategy of anticancer drugs, in combination with natural substances, is an emerging platform for the improvement of drugs with chemotherapeutic potential, the reversing of MDR, and the reduction of the side effects of chemotherapy [60].

This mixed micellar system was prepared through a solvent diffusion method by mixing 1,2-distearoyl-sn-glycero-3-phosphoethanolamine-N-[methoxy(polyethylene glycol)-2000] (DSPE-PEG2000) with the copolymer polyethylene glycol-poly (ε-caprolactone) (PEG-PCL); previously synthesized by a cationic ring-opening of caprolactone using MeO-PEO5K as a macroinitiator. Such mixed micelles are characterized by their excellent biocompatibility, low toxicity profile, and sustained release of anticancer drugs [59]. The micellar system showed better encapsulation efficiency for PTX compared to NRG, probably due to differences in their hydrophobic nature. In addition, they showed adequate structural characteristics to achieve deep tissue penetrations at the target sites of the body. Cellular uptake and cytotoxicity studies on human breast cancer MCF-7 cells confirmed the enhanced anticancer activity of PTX when coadministered with NRG encapsulated in mixed polymeric micelles. According to the authors, these improvements may occur due to the presence of NRG, which could increase the cellular uptake of PTX or inhibit P-gp-mediated efflux in MDR cells. It could also be attributed to the size and composition of the micellar system used, which, by containing hydrophilic poly(ethylene oxide), blocks increases PTX internalization or reverses P-gp-mediated efflux in MDR cancer cell lines.

Lavrador et al. (2018) developed NRG-loaded polymeric micelles as an approach to modulate the osteogenic differentiation of stem cells [44]. NRG is reported as one of the most promising phytotherapeutics that can potentially enhance the proliferation and differentiation of osteoprogenitor cells into osteoblasts and, at the same time, inhibit osteoclastic activity. The polymeric micelles designed were synthesized via a Michael-type addition reaction between hydrophilic methoxy-poly(ethylene glycol)–maleimide (mPEG-MAL) and hydrophobic thiol–poly(l-lactide) (PLA-SH). Subsequently, the copolymer and NRG were self assembled by nanoprecipitation. These NRG micelles show a high EE with a suitable size (84.48 ± 2.44 nm) for possible parenteral administration to bone tissue, a negative-charged surface, and a sustained release profile of NRG. The stability studies revealed high stability of nanomicelles for two weeks at 4 °C, both in deionized water and a PBS buffer at pH = 7.4. In vitro results confirm that the formulated micelles have the potential to be internalized within human adipose-derived stem cells (hASC), with trafficking to the lysosomal/endosomal compartments. They also reveal that upon internalization of the micellar system, NRG enhances its pro-osteogenic properties. In particular, micellar NRG induces higher osteopontin expression, significantly increases the ALP activity, and enhances matrix mineralization in these cells compared to free compound administration.

### 5.3. Polymer-Based Nanoparticles

Polymer-based nanoparticles can be considered one of the most evaluated drug delivery systems for NRG loading, mainly due to the versatility offered by the different polymers used, as well as the variety of the preparation techniques employed [61]. These particles are made up of natural or synthetic polymers and, in practice, they include structures up to 300–400 nm in size. Within the natural polymers, Malathy and Iyer (2018) developed NRG-loaded chitosan (CN) nanoparticles (NRG-CNs) as potential formulations for bone regeneration [62]. These NRG-CNs were synthesized by an ionic gelation technique using sodium-tripoly phosphate (TPP) as a crosslinker agent. The reported results show that the average size of the nanoparticles prepared with 0.5% CN + 0.1% TPP remained below 100 nm, even after loading the NRG (93 nm). However, it is important to control the crosslinking since, when increasing the percentage of TPP to 0.25%, a considerable increase in size is evident (250 nm). Although the authors themselves point out that the report is a preliminary in vitro study, and does not highlight the quantitative advantages of the developed formulation over free NRG for the proposed objective, the results expose that the NRG loaded in the CN nanoparticles is capable of promoting the secretion of bone formation proteins (BMP), in addition to improving the proliferation and osteogenic differentiation of BMSCs. On the other hand, they also demonstrate that this formulation has the ability to inhibit both the proliferation of cancer cells in vitro and albumin denaturation, as well as triggering the development of adequate antioxidant activity even better than free NRG.

Ebrahimi et al. (2020) also designed nanoparticles to load NRG from biocompatible natural polymers such as CN. However, in this case, the nanoparticles are part of a more complex formulation (hydrogel) to be used as a potential nanocomposite biomaterial for the regeneration of peripheral nerves [63]. The neuroprotective effects of NRG in the prevention or treatment of neurological disorders have been described. In these studies, NRG and berberine (BER) were independently encapsulated in CN nanoparticles synthesized based on ion gelation methods. The results show that the CN-NPs loaded with NRG and BER have a large average size of 636 nm and 594 nm respectively, and both have a positive zeta potential. The relatively low EE achieved, of around 50%, is attributed by the authors to the positive electrical charge of the Cs polymer and the cationic charge of BER and NAR, which limit the obtainment of higher levels. The hydrogels containing the nanoparticles were prepared by mixing solutions of CN cross linked by β-glycerol phosphate (β-GP) with solutions of alginate cross linked by CaCl_2_. Finally, BER/NRG-NPs powders were added into the polymeric solution with ratios of 1% and 10% weight of the polymer, respectively. In vitro viability studies evidence positive effects of both compounds on the proliferation of PC12 cells compared to the control group. In addition, the authors demonstrate in animal studies that treatment with hydrogels significantly promotes nerve regeneration. In their conclusions, they describe that treatment of damaged nerves using CN/alginate hydrogels containing NRG and BER-loaded NPs resulted in the recovery of both sensory and motor functions (sciatic index function, and anatomical healing).

Imam et al. (2022) formulated hybrid nanoparticles to work as oral nanocarriers for NRG delivery (NRG-HN) using a biodegradable lipid (soy lecithin), CN, and polyethylene glycol D-α-tocopheryl succinate (TPGS), which fulfilled the role of a surfactant for the preparation [64]. According to the authors, the use of TPGS as a surfactant increases the solubility of NPs when conjugated with chitosan, which also enhances the stability of the hybrid nanoparticles. This helps in the preparation of NPs due to the electrostatic attraction between the negative charge of lecithin and the positive charge of CS. The results show that the three formulation variables, that is, the lipid, polymer, and surfactant content used in the preparation significantly influence the particle size and the EE. In general, the higher the concentration of lipid, polymer, and surfactant, the larger the nanoparticles and the higher the EE% obtained. The optimized formulation (NRG-HNop) presents a particle size of 246 ± 8 nm with a narrow size distribution (PDI = 0.23) and an EE of NRG of 84 ± 2%. The in vitro release profile of NRG-HNop exhibits an NRG release of about 70% after 12 h and 90% after 24 h. Based on the results reported, it is evident that this formulation improves the permeability (3.7 times) of NRG through a goat intestinal membrane compared to free NRG, significantly improves its antimicrobial activity, especially against *Escherichia coli* (Gram-negative), enhances the antioxidant activity of NRG at all concentrations tested, and demonstrates greater effectiveness in killing breast cancer cell lines (MCF7) compared to free NRG. According to the authors, the presence of the chitosan polymer in the nanoparticles may be contributing to the greater antibacterial activity as well as to the anticancer activity. Although this report focuses on the preparation and characterization of NRG hybrid nanoparticles as NRG nanocarriers, it is suggested that this formulation could be an alternative method for oral administration to treat cancer cells. Nallamuthu et al. (2020) fabricated colloidal zein/sodium caseinate nanoparticles (NZS) loaded with NRG to enhance the activity of this bioactive in the management of disorders related to obesity [65]. Zein is a prolamin protein obtained from the endosperm of maize (Zea mays L), extensively studied for the encapsulation of hydrophobic molecules due to its ability to self assemble into spherical colloidal nanoparticles with a hydrophobic core. Casein sodium salt from bovine milk is a phosphoprotein used as an emulsifier and stabilizer for foods. The optimized formulation reports spherical particles of 234 nm mean size with a monodisperse size distribution (PDI = 0.213), negative zeta potential (−28.2 mV), and an EE of 71 ± 2%. The differential scanning calorimetry results suggest that these nanoparticles are thermally stable and therefore suitable for the encapsulation of thermosensitive molecules. NRG release profiles from nanocomposite nanoparticles, under simulated gastric and intestinal pH conditions for 72 h, show an initial rapid release followed by sustained release, pH-dependent. Under the influence of intestinal pH, the release rate of NRG was found to be higher, reflecting a higher in vitro bioaccessibility compared to the acidic gastric condition which may contribute to increased absorption into the systemic circulation. The authors report that NRG formulated in zein/caseinate biopolymers nanoparticles significantly enhances its antiadipogenic activity on murine 3T3-L1 preadipocytes by reducing lipid accumulation, in addition to exhibiting a better antioxidant activity compared to free NRG. The research findings demonstrate that Zein-NaCas copolymers are promising carriers for antiadipogenic NRG delivery, with the potential for application in various healthy foods.

Another natural polymer used to encapsulate NRG is β-cyclodextrin (β-CD). This is a biocompatible polymer belonging to the group of carbohydrates (cyclic oligosaccharides), soluble in water and with a hydrophobic central cavity capable of containing compounds poorly soluble in water and transported in aqueous solutions. Hussain et al. (2022) prepared NRG-loaded β-CD nanoparticles via the solvent evaporation method a as potential nanoformulation to improve the efficacy of this compound against bacterial infections [66]. The reported results show spherical NRG NPs of 70 nm in mean diameter, with a uniform size distribution, net negative surface charge, and high EE. Thermogravimetric analysis reveals that encapsulation in β-CD nanoparticles significantly increases the thermal stability of NRG. The in vitro release profile of NRG from β-CD nanoparticles suggests that the formulation has better compatibility with intestinal pH for the release of this bioactive. Regarding the bactericidal potential of NRG, reported microbiological assays (MTT) and three-dimensional morphological analysis (AFM) show that NRG loaded into β-CD nanoparticles significantly increased its antibacterial activity, causing prominent destructive changes in the morphology of the different bacterial cells tested. These findings allow the authors to suggest that this formulation may be a successful alternative to enhance NRG efficacy against various bacterial infections.

Mohanty et al. (2021) reported that polymeric nanoparticles (NPs) made from the synthetic polymer poly-co-glycolic acid (PLGA) tend to improve the oral efficiency of NRG and thus enhance its beneficial effects in osteoarticular degenerative disorders such as rheumatoid arthritis (RA) [67]. PLGA polymer is a biocompatible and biodegradable amphipathic copolymer, widely used for the manufacture of nanoparticles in order to improve the solubility, pharmacokinetics, and stability of encapsulated drugs. These NRG-loaded PLGA nanoparticles were fabricated by a modified solvent emulsification—evaporation technique using acetone—ethanol (2:1) as a solvent system in combination with stabilizers such as Pluronic F68 and sodium deoxylate, along with a 2% PVA solution. According to the reported characterization studies, the optimized formulation (FN4) has a small particle size of 179.7 ± 2.1 nm with a low PDI (0.206), a negative zeta potential, and an EE on the order of 70%. The lyophilized formulation proved to be highly stable under different storage conditions and provided a higher release of NRG (82.11 ± 3.65%) in phosphate buffer pH 6.8 for 24 h with a sustained effect. Ex vivo permeation analysis carried out through a goat intestinal membrane indicated 80% NRG release in 24 h. Studies conducted in vivo in a chronic arthritic model (FCA) induced in rats allowed the authors to demonstrate that the administration of NRG loaded-polymeric nanoparticles produces a greater reduction in the severity of arthritis in FCA than those treated with NRG alone The report indicates decreased levels of proinflammatory cytokines (INF-γ, IL-6, and TNF-α) with an increase in IL-10 (anti-inflammatory cytokine), and the attenuation of serum rheumatoid factor (RF-factor) levels and C-reactive protein (CRP) compared to nanoparticles blank and NRG alone. As a result of their research, the authors postulate this formulation as a suitable approach for delivering the bioactive NRG to the intestine, avoiding the formula’s gastric degradation and improving its bioavailability. Wang et al. (2022) also encapsulated NRG in PLGA nanoparticles by the emulsion–diffusion–evaporation method for potential applications in the food industry and the field of nanomedicine [68]. This nanoformulation, defined by the authors as NRG loaded-nanospheres, showed a mean diameter of 137 ± 30 nm with an EE of the flavonoid of 86.4%. The compound is also to have an initial burst effect in the initial 24 h and followed by sustained release lasting for 10 days. The authors highlight that their formulation exhibits strong antibacterial activity against *E. coli* and *S. aureus* at a low concentration of NRG (0.2 mg/mL), as well as an important inhibitory effect against human esophageal cancer cells (ECA 109), which could be useful to improve human health or the bioavailability of food components and pharmaceuticals.

### 5.4. Lipid Nanoparticles

Lipid nanoparticles (LNPs) are nanostructures composed of lipids widely used as carriers for a variety of therapeutic agents, from biotechnological products to small molecules, mainly due to the fact that they are easy to produce, nontoxic, and biocompatible formulations. Solid lipid nanoparticles (SLPs) [69] can be also considered to be nanostructured lipid carriers (NLCs), called second-generation lipid nanoparticles [70,71,72].

Zhu et al. (2020) designed an NRG-loaded NLC containing a liquid lipid such as coix seed oil (CSO) working as a potential formulation against hepatocellular carcinoma (HCC) [73]. The authors postulate that CSO can enhance the loading efficiency of NRG and exert synergistic two-drug antitumor activity. The NRG-loaded NLCs exhibit a spherical morphology of small size (38.77 ± 0.25 nm) with a uniform size distribution (PDI = 0.29), slightly negative zeta potential, and an EE greater than 90%. The small size obtained would have the potential to penetrate deeply into the tumor.

Although the in vitro release tests (pH 5 and 7.4) evidence a faster release of NRG from NLCs prepared with CSO, compared to other NLCs prepared with conventional liquid lipids (oleic acid and neodecanoate triglycerides), the formulation of NLC with CSO maintains the capacity to release NRG in a sustained manner for 36 h. In vitro cytotoxicity assays using CCK-8 as a staining method for viable cells and Annexin V-PE assay kit for determination of apoptosis shows a greater antiproliferative effect on HepG2 cells, and a significantly greater capacity to induce apoptosis of the formulation containing NRG and CSO compared to the other formulations tested. Furthermore, this dual formulation was more effective in suppressing tumor growth in a xenograft model of hepatocellular carcinoma, exhibiting also increased spleen weight and significantly higher levels of IL-6 and IL-10 in the serum of the mice. These positive findings allow the authors to suggest that this novel lipid formulation could play an important role in the effective delivery of two anticancer agents. Alhalmi et al. (2022) also prepared and optimized NLCs for the codelivery of NRG and an estrogen receptor modulator (raloxifene hydrochloride (RLX)) as an alternative formulation for breast cancer [74]. An optimized dual nanostructure prepared with Compritol 888 ATO and oleic acid by a hot homogenization sonication method, shows small, almost spherical particles (137.12 nm) with a homogeneous size distribution (0.266), positive zeta potential, and high EE for both compounds. Although the authors do not report results referring to the antitumor activity of the optimized formulation, the different in vitro studies reported show that the NLCs developed are capable of releasing both compounds at a sustained rate for 24 h (pH 6.8 buffer) with an initial burst release; improving the ex vivo intestinal permeability profiles of both NRG and RLX versus an NRG/RLX suspension; and increasing antioxidant activity in vitro (2,2-Diphenyl-1-picrylhydrazyl, DPPH) compared to individual compounds, which suggests the exertion of a synergistic antioxidant effect. Furthermore, this proves to be a safe formulation and its lyophilized form maintains satisfactory stability and integrity for three months. The authors previously reported the same NLC optimized for NRG-only loading with similar results in terms of sustained release profile and increased intestinal permeation capacity ex vivo compared to an NRG suspension, demonstrating the promising potential of this formulation to improve the oral bioavailability of NRG [75].

### 5.5. Other Formulations

Other nanoformulations that are essentially used to improve water solubility and provide a more stable delivery system for NRG are described below. Results from the literature are summarized in Table 1.

## 6. Conclusions and Future Perspectives

Natural products have historically contributed significantly to pharmacotherapy. NRG represents a potential therapeutic natural compound capable of preventing, controlling, and/or reversing different physiological disorders. In recent years, there has been an exponential growth in the number of investigations related to NRG, describing its biological properties and its beneficial implications for human health.

Unfortunately, as it occurs with other compounds, the therapeutic use of NRG is substantially limited to the drawbacks mentioned above.

For this reason, it is essential for pharmaceutical technology to accompany and support the expansion of research on this compound and make it possible to overcome its physicochemical limitations in order to enhance its therapeutic efficacy and advance toward clinical application.

## Figures and Tables

**Table 1:** Other nanoformulations designed for the delivery of Naringin.

Type of Formulation	Features	Preparation Method/Technique	Application	Highlights	Reference
TiO_2_ nanotubes	Chitosan-coated NRG-loaded TiO_2_ nanotubes	TiO_2_ nanotubes were fabricated by electrochemical anodization Then, NRG was loaded into TNTs by direct dropping and coated with chitosan layers.	* Osteogenesis	* The controlled release of NRG showed a burst release (51%) during the first 48 h of immersion, and a maximum release at 72 h. * Chitosan-coated NRG-loaded TiO_2_ nanotubes enhanced osteoblast spreading, proliferation, alkaline phosphatase activity, and late-stage osteoblast mineralization.	[76]
Rutile (TiO_2_) nanorod films	NRG mixed with gelatin methacryloyl (GelMA) hydrogel incorporated into Rutile nanorod films	NRG was loaded in two distinct manners in GelMA hydrogel (mixing and soaking) and subsequently incorporated on TiO_2_ nanorod coatings.	* Osteogenesis	* The size of NRG loaded-nanorods was nearly 600 nm. * The release kinetics of two-load hydrogel coating was different. * The NRG-loaded coatings facilitated the adhesion, proliferation, late differentiation, and mineralization of MSCs.	[77]
Metal–organic frameworks (MOFs) nanocrystals	NRG-loaded multifunctional mineralized Collagen coating with the aid of MOFs nanocrystals	* The MOFs nanocrystals were synthesized by hydrothermal method. * Mineralized collagen coatings were deposited on a metal titanium surface by electrochemical deposition.	* Osteogenesis and antibacterial activity	* The products exhibited a monodispersed spherical morphology with diameters ranging from 450 to 600 nm. * The formulation significantly improved attachment, osteogenic proliferation, differentiation, and mineralization of mesenchymal stem cells (MSCs). * Col/MOF/NRG substrates showed the best activity in preventing *S*. *aureus* proliferation compared to other substrates.	[78]
Microspheres	NRG-loaded sodium alginate microspheres incorporated into brushite to prepare composite scaffolds	Complex multi-step method.	* For bone tissue engineering	* The particle size of the microspheres was mainly distributed from 300 to 600 μm. * The composite showed good degradability and drug-release ability * The loading of pyrite and NRG simultaneously at a certain dosage promoted mineralization ability and enhanced the expression of alkaline phosphatase of osteoblasts	[79]
Microstructured titanium (Micro-Ti)	Micro-Ti covered with NRG, chitosan, and gelatin multilayers	* Micro-Ti was prepared on titanium surfaces by dual acid etching. * Micro-Ti was covered with NRG, chitosan, and gelatin multilayers through a layer-by-layer technique.	* Osteogenesis in osteoporosis patients	* Microstructured titanium functionalized by NRG-inserted multilayers, enhanced osteoblast differentiation, and inhibited osteoclast formation.	[80]
Microsphere/SAIB hybrid depots	NRG-loaded microsphere/ sucrose acetate isobutyrate (Ng-m-SAIB) hybrid depots	Microspheres were prepared using a single-nozzle electro-spraying setup. Then, NRG-microspheres were dispersed into the SAIB solution by vortexing for 5 min to prepare NRG-m-SAIB depots.	* Bone regeneration	* Osteoblast-microsphere interactions were better when the NRG content was 4%. * Loading NRG microspheres into SAIB depots drastically reduced burst release, with a sustained and continuous release until day 61. * The highest NRG EE in the microspheres was 64.3%. *After 8 weeks of healing of the bone defect, the group treated with this formulation exhibited better bone formation with BV/TV reaching 53.1%.	[81]
Microspheres encapsulated in a scaffold	NRG-loaded gelatin microspheres encapsulated in a nanohydroxyapatite/silk fibroin scaffold (NRG/GMs/nHA/SF)	* Gelatin microcapsules were fabricated using an emulsion solvent evaporation method. * nHA/SF composite scaffolds with NRG-loaded GM microcapsules were fabricated by a multi-step process.	* Bone tissue engineering * Critical-size vertebral defects	* NRG/GM/nHA/SF scaffolds exhibited good biocompatibility, biomechanical strength, and promoted BMSC adhesion, proliferation, and calcium nodule formation in vitro. * NRG/GMs/nHA/SF scaffolds showed greater potential for osteogenic differentiation than other scaffolds in vitro. * In vivo, gradual new bone formation was observed, and bone defects recovered by 16 weeks.	[82]
Microspheres	Silk fibroin (SF)/hydroxyapatite (HAp) scaffolds inlaid with NRG loaded poly lactic-co-glycolic acid (PLGA) microspheres	* PLGA microspheres incorporated with NRG were prepared by the w/o/w emulsion solvent evaporation method. * PLGA microspheres containing NRG (5 mg/mL) were loaded into the suspension to form MSN/SF/HAp scaffolds. The suspension was freeze-dried and then they were treated with methanol to induce β-sheet formation.	* Bone tissue engineering	* The mean diameter of MSN/SF/Hap was 99.4 ± 3.6 μm. * The EE was 78.5 ± 3.6%. * In vitro release profile of NRG from PLGA microspheres and MSN/SF/HAp scaffolds was approximately 83.9% and 71.9%, respectively, after 36 days of incubation. * In vivo analysis indicated that MSN/SF/HAp promotes the repair of bone defects.	[83]
Microparticles	NRG and NRGN gastro-resistant microparticles using cellulose acetate phthalate (CAP) as the coating polymer	NRG and NRGN gastro-resistant microparticles were formulated by spray-drying technique.	* Controlled drug release to the intestine	* 2% CAP solutions in an aqueous buffer at pH 7.5 were the most efficient in drug coating. * The particle sizes ranged from 3 to 6 μm. * The microparticles showed a pH-dependent biphasic in vitro release profile, capable of protecting the flavonoids in the gastric environment and releasing them in the intestinal tract.	[13]
Dry powder microparticles	NRG dry powder with added Amino Acids (AA)	Different NRG-dried powders were manufactured by cospray drying.	* Cystic fibrosis therapy * To treat lung intrinsic inflammation and prevent tissue damage in CF patients	* Very interesting results were obtained in terms of fluidity and aerodynamic performance using leucine, histidine, and proline. * N-leu and N-pro powders showed a size within a range of 2.75–3.42 µm. * Leucine cospray-dried with the NRG improved both the aerodynamic properties and in vitro pharmacological activity of NRG.	[84]
Nanoparticles	NRG-linoleic acid prodrugs nanoassemblies	Covalent conjugation of NRG with linoleic acid by impulsively nanoassembly using DIEA as a crosslinker.	* Lung cancer	* The particle size of nanoassembly NRG-NPs was 82.7 ± 2.1 nm. * NRG-NPs demonstrated a sustained release of NRG after 7 days of incubation and increased cellular uptake efficiency in lung cancer cells. * In vitro cytotoxicity activity showed NRG-NPs induced apoptosis in human lung cancer cells.	[85]
Ointment formulation	Soft paraffin-based cream containing 1, 2, and 4% (*w*/*w*) NRG	NRG ointment for topical application was prepared by a previously described method [86,87]	* Wound healing	* Treatment with NOF (2 and 4%, *w*/*w*) significantly decreased the wound area and epithelialization period, increasing the rate of wound contraction. * NOF significantly restored the expression of inflammatory (NF-jB, TNF-a, and IL), apoptotic (pol-g and Bax) mediators, and growth factors (VEGF and TGF-b) * NOF restored histological alterations in the wound skin.	[88]
Hydrogel	NRG-loaded hydrogel polymerized sodium alginate/bioglass thermo-responsive	Hydrogels were prepared by adding agarose solutions (2%) to assure drug loading with a combination of Sodium alginate (SA) solution, gluconolactone, and bioglass powder (BG).	* Reconstruction of the articular cartilage	* The hydrogel showed that NRG stimulated chondrocyte proliferation with a concentration of 10 μM for three consecutive days. * NRG-BG hydrogels maintained normal chondrocyte morphology, promote macrophage polarization into M2 types, effectively inhibit ECM degradations, and restore defective tissue cartilage.	[89]
Hydrogel	NRG-loaded Arabic gum (AG)/pectin hydrogel	The hydrogel was prepared by adding an 8 M CaCl_2_ solution as a crosslinking agent to a mixture of pectin (8%, *w*/*v*) and AG (8%, *w*/*v*) solutions with constant stirring. It was subsequently lyophilized and stored in a desiccator. The lyophilized hydrogel was added to a solution of NRG (30% ethanol) under sonication. NRG loading was performed by adding the lyophilized hydrogel to an NRG solution (30% ethanol) under constant agitation for 24 h.	* Wound healing	* NRG hydrogel was able to accelerate wound healing in terms of enhanced angiogenesis, re-epithelialization, and collagen deposition. * NRG hydrogel significantly downregulated the mRNA expression of inflammatory mediators (TNF-α) and apoptosis (BAX). * NRG hydrogel demonstrated potent antioxidant activity.	[90]
Nanoparticles entrapped in biodegradable three-dimensional scaffolds	NRG loaded-bovine serum albumin nanoparticles entrapped in porous polycaprolactone Scaffold (PS-NRG-BSANPs)	PS-NRG-BSANPs were engineered by solvent casting and particulate leaching methods.	* Bone tissue engineering	* The results indicated that no chemical modification of NRG occurs throughout the manufacturing process. * The release profile of NRG from PS-NRG-BSANPs showed sustained release for 12 days. * The PS-NRG-BSANPs scaffold enhanced calcium deposition and collagen matrix formation under osteogenic conditions with the C3H10T1/2 cell line.	[91]

## Data Availability

Not applicable.
